# A Soft and Skin-Interfaced Smart Patch Based on Fiber Optics for Cardiorespiratory Monitoring

**DOI:** 10.3390/bios12060363

**Published:** 2022-05-26

**Authors:** Daniela Lo Presti, Daniele Bianchi, Carlo Massaroni, Alessio Gizzi, Emiliano Schena

**Affiliations:** 1 Unit of Measurements and Biomedical Instrumentation, Department of Engineering, Università Campus Bio-Medico di Roma, Via Alvaro del Portillo, 00128 Rome, Italy; d.lopresti@unicampus.it (D.L.P.); c.massaroni@unicampus.it (C.M.); 2 Unit of Nonlinear Physics and Mathematical Models, Department of Engineering, University of Rome Campus Bio-Medico, 00128 Rome, Italy; d.bianchi@unicampus.it (D.B.); a.gizzi@unicampus.it (A.G.)

**Keywords:** cardiorespiratory monitoring, FBG technology, soft sensors for respiratory and cardiac monitoring, biosensors for vital signs monitoring

## Abstract

Wearables are valuable solutions for monitoring a variety of physiological parameters. Their application in cardiorespiratory monitoring may significantly impact global health problems and the economic burden related to cardiovascular and respiratory diseases. Here, we describe a soft biosensor capable of monitoring heart (HR) and respiratory (RR) rates simultaneously. We show that a skin-interfaced biosensor based on fiber optics (i.e., the smart patch) is capable of estimating HR and RR by detecting local ribcage strain caused by breathing and heart beating. The system addresses some of the main technical challenges that limit the wide-scale use of wearables, such as the simultaneous monitoring of HR and RR via single sensing modalities, their limited skin compliance, and low sensitivity. We demonstrate that the smart patch estimates HR and RR with high fidelity under different respiratory conditions and common daily body positions. We highlight the system potentiality of real-time cardiorespiratory monitoring in a broad range of home settings.

## 1. Introduction

The monitoring of cardiorespiratory activity offers crucial information for preventing critical health conditions, thus promoting the early diagnosis and treatment of cardiovascular and respiratory diseases (CVRDs) [1]. CVRDs such as heart attacks, stroke, chronic obstructive pulmonary diseases, and asthma are a growing cause of morbidity and mortality worldwide and represent high clinical and economic burdens [2,3,4]. These diseases account for the deaths of 22 million people annually, including elderly people, but also children and adults [1,5].

The heart rate (HR) and respiratory rate (RR) have central roles in assessing cardiorespiratory functionalities since an early detection of abnormal values may indicate serious cardiorespiratory diseases [6,7,8,9]. The term “abnormal” refers to non-physiological HR and RR values established at rest. In the literature, several studies underlined the clinical relevance of resting HR for the mortality evaluation, prediction, and prognosis of cardiovascular diseases [10,11]. Similarly, RR measurements at rest or while sleeping have been considered clinically relevant for respiratory disorders and heart failure prediction [12,13,14]. To date, the gold standard for HR monitoring is the electrocardiogram (ECG) [15], whereas RR is commonly monitored by impedance pneumography [14]. These methods are used to monitor cardiac and respiratory parameters in clinical settings or during hospitalization, showing some issues in the early detection of symptoms, often subtle when they first appear [16]. Recently, interest has grown to open new frontiers in health monitoring thanks to the Internet of Things (IoT) revolution [17]. In this area, new devices constantly available and wearable have been designed to track human health in home settings [18,19,20]. The increasing adoption of these devices has fostered substantial progress in wearable technology for cardiorespiratory monitoring [21]. Among others, strain sensors such as resistive [22], capacitive [23], and fiber Bragg grating (FBG) sensors [24] have been placed on garments to detect local chest deformations due to breathing and heart beating [25,26,27,28]. However, most of these systems showed the best performance in RR monitoring [29,30]. Regarding the HR estimation, strain sensors for cardiorespiratory monitoring demonstrated a good capability in detecting cardiac-induced chest deformations during breath-hold stages (when the respiratory contributions are discharged automatically). Their performance decreases when HR is extracted during breathing [31,32].

Acoustic sensors such as microphones on the thorax have also been proposed for cardiorespiratory monitoring, but the interference between respiratory and heart sounds complicates the simultaneous estimation of RR and HR [33,34]. Other studies proposed inertial sensors, such as accelerometers and gyroscopes, placed on specific landmarks of the chest (e.g., xiphoid process and left sternum) to detect heart-induced accelerations (i.e., generally known as seismocardiogram—SCG—signals) and angular velocities (i.e., since 2015 referred to as gyrocardiogram—GCG—signals) originating on the chest surface [35,36]. Recently, this technology has also been proposed for cardiorespiratory monitoring, but it does not ensure yet high measurement accuracy, and the comfort is limited by its rigidness and low compliance with the skin [30].

The latest technological advancements in material science and mechanics are bringing new opportunities for wearables and addressing many of these shortcomings. A new class of wearable skin-interfaced sensors with the characteristics of lightness, flexibility, and portability have found powerful applications in non-invasive physiological monitoring [37,38]. Examples of those innovative technologies include ultra-thin and stretchable biosensors such as e-tattoos and patches with excellent compliance with the skin curvatures and body movements to provide additional patient comfort, skin safety, and signal accuracy [24,35,39,40,41].

FBG-based biosensors have recently attracted considerable attention since they combine the advantages of FBG technology such as small cross-sectional area (an optical fiber is not larger than a human hair), biocompatibility, electromagnetic interference immunity, and high sensitivity with the properties of a hosting material [24,32,42,43]. This approach improves the FBG brightness and poses an exciting option for the design of high-performance flexible wearable devices. Indeed, their structural and functional behaviors may be tunable by the matrix shape, the casting material, and the sensor positioning into the hosting structure. A few studies have proposed FBG-based wearable biosensors for cardiorespiratory monitoring showing the best performance in RR monitoring [24,44,45]. No deeper investigations for measuring HR during breathing stages and under various body postures have been carried out.

Here, we report the design, fabrication, and feasibility assessment of a novel skin-interfaced biosensor (referred to as smart patch) based on FBG technology to simultaneously monitor RR and HR. The system performances were investigated under three body postures to deeply assess their influence on the smart patch capability of monitoring both RR and HR. The small form-factor, the single sensing modality, and the skin-like properties are the main distinctive features of the proposed solution. To the best of our knowledge, this is the first skin-mountable smart patch based on FBG for cardiorespiratory monitoring. In the following sections, we firstly describe the working principle and the fabrication of the proposed biosensor designed to enable the transduction of chest deformations into an optical signal thanks to an effective skin-device coupling. Then, we present a model simulation to prove the effectiveness of the chosen design in concentrating the maximal strain on the FBG sensor encapsulated into the patch. Finally, we evaluate the metrological properties of the proposed biosensor, and we perform the feasibility assessment on healthy subjects by investigating the system performance in the simultaneous RR and HR monitoring in supine, sitting, and standing positions.

## 2. The Smart Patch Working Principle, Computation Model, and Fabrication

### 2.1. The Smart Patch Working Principle

An FBG sensor is made of distributed Bragg reflectors in a short segment of optical fiber that reflects a specific wavelength, the so-called Bragg wavelength (λ_B_). The term grating refers to a periodic change in the core’s refractive index. When the light travels inside the grating structure, a portion of the light is reflected back from each grating plane once the Bragg condition is met. The Bragg condition is given by:

(1) $$\lambda_{B}=2{\Lambda \eta }_{\mathrm{eff}}$$

where Λ is the grating period (i.e., the distance between two consecutive grating planes) and η_eff_ is the effective refractive index of the fiber core.

The principle of wavelength shift is at the basis of the FBG sensing mechanism. As described by the Bragg condition, λ_B_ depends on two physical parameters: Λ and η_eff_. A change in these parameters causes a shift of λ_B_ (Δλ_B_) either to the left (if compressed/cooled) or to the right (if strained/warmed). The value of Δλ_B_ depends linearly on the amount of external perturbation applied to the FBG by the following:

(2) $${\Delta \lambda }_{B}=K_{\varepsilon }\Delta \varepsilon +K_{T}\Delta T$$

with the strain sensitivity $K_{\varepsilon }=\left(1-p_{e}\right)\lambda_{B}$, the thermal sensitivity $K_{T}=\left(\alpha +\eta \right)\lambda_{B}$, $p_{e}$ as the photo-elastic coefficient, α as the thermal expansion coefficient, and η as the thermos-optic coefficient.

**Figure 1:**
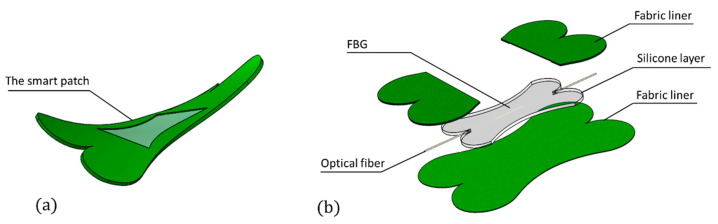
(**a**) The smart patch and (**b**) its multi-layered structure.

In this study, an FBG sensor was encapsulated into a flexible matrix (i.e., silicone layer) and then layered between two fabric liners to obtain the proposed smart patch. Figure 1 shows the 3D model of the smart patch with its multi-layered structure.

The FBG encapsulation into flexible substrates was proposed to confer a skin-like softness and appearance to the sensor and improve the user acceptability. Thanks to the intrinsic $K_{\varepsilon }$ of the FBG sensor and the proper skin adherence of the patch, the proposed system was used to monitor the RR and HR from chest wall deformations. Indeed, when the chest wall expands and contracts due to breathing and heart beating, periodic perturbations occur on the patch surface. These perturbations are transduced into ε at the skin–polymer interface and transferred to the encapsulated FBG, causing a Δλ_B_, accordingly.

**Figure 2:**
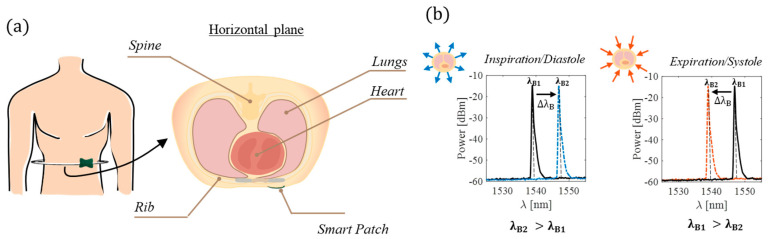
The smart patch working principle during breathing/heart beating: (**a**) the sensor placement and (**b**) the spectrum changes during inspiratory/diastolic (from black to blue line) and expiratory/systolic (from black to orange line) phases.

Figure 2 shows the smart patch working principle during breathing and heart beating. The volumetric chest expansion caused by the air inflection induces a ε on the patch surface, leading to a right Δλ_B_ (from λ_B1_ to λ_B2_); the volumetric contraction caused by the air deflection otherwise induces a compression on the patch and, in turn, a left Δλ_B_. The perturbations caused by the heart beating are smaller than the ones caused by the breathing activity and challenging to model. In a simplified way, for every beat, the heart contracts and expands with rhythmic activity and the apex taping against the chest wall. The heart, during systole, generally withdraws from the chest wall except for the apex.

The effect of this withdrawal can be observed on the chest wall as an inward movement dampened by the bone and tissue structures that causes a left Δλ_B_. Otherwise, during the diastolic phase, the heart chambers fill in, the systolic impulse recedes, and the chest wall expands itself with a consequent right Δλ_B_ [46].

### 2.2. In Silico Model

An in silico computational model was developed to prove the effectiveness of the patch design. The model geometry consists of the CAD structure (designed in SOLIDWORKS^®^) matching the sensor design. Once patched to the skin surface, the sensor geometry was prescribed with bending and stretching boundary conditions. Deformations and vibrations were applied to the skin to model the simultaneous activity of the heart and lungs. The finite element mechanical analysis was carried out by employing the software COMSOL Multiphysics^®^ (COMSOL Inc., Stockholm, Sweden): the computational domain comprehends the patch, composed of silicone matrix and the fabric liners, attached to the human skin modeled as a layer with a constant thickness of 5 mm. The patch and the skin material are considered linear-elastic and isotropic, characterized by the Young modulus (E) and Poisson coefficient (ν).

All the model parameters are summarized in Table 1. We implemented elastic material models featuring a E of 4.5 MPa for the silicone layer and a E of 0.02 MPa for the fabric liner [47]. An additional substrate modeled the skin surface (E = 0.13 MPa) in contact with the smart patch [48]. For all the structures, a ν of 0.49 was employed, replicating the quasi-incompressibility of the rubber-like materials.

**Figure 3:**
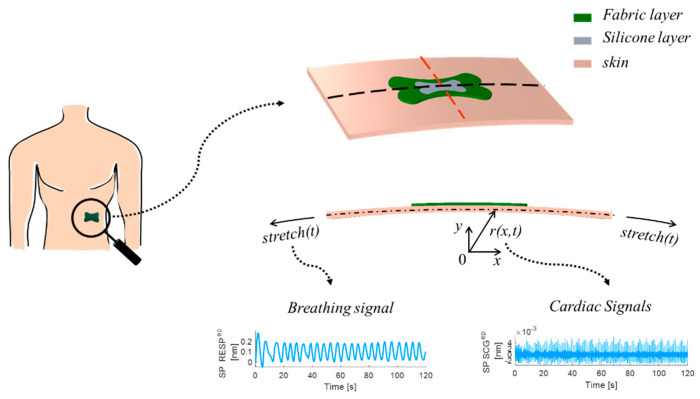
Schematic view of the computational model: on the left, the position of the sensor on the body; on the right, the detail of the computational domain with the boundary conditions used in the numerical analysis.

The finite element numerical analysis addresses the stretch and the bending of the skin during the breath, accounting for cardiac activities. Model geometry and the boundary conditions are depicted in Figure 3.

**Figure 4:**
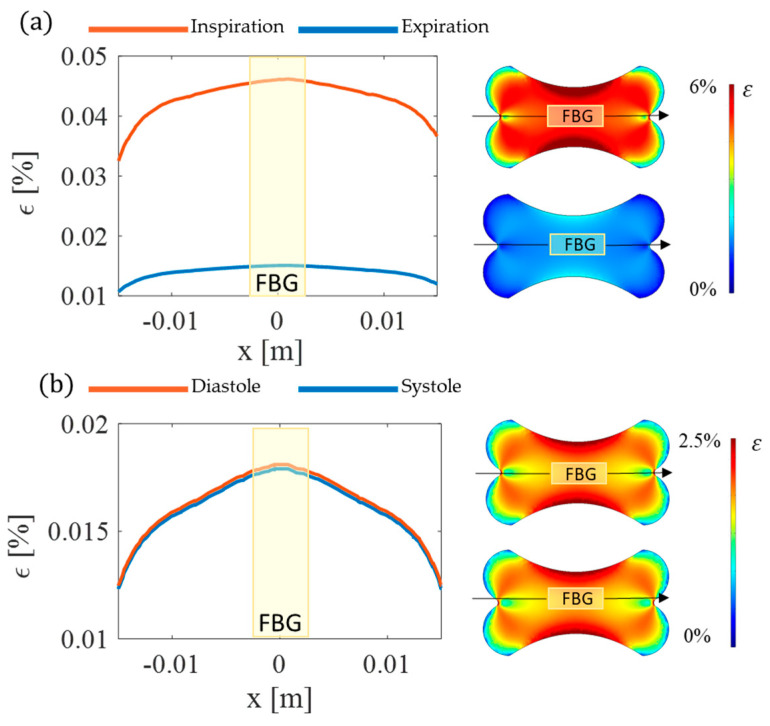
Summary of computational studies highlighting (**a**) the effects of breathing (blue line refers to inspiration and orange line to expiration) and (**b**) the effects of heart beating (blue line refers to systole and orange line to diastole) on the optical fiber and the FBG sensor.

A radial function r(x,t) is defined to model the shape of the chest and the cardiac activities. Aiming to check the soundness of the patch design, the results are shown in terms of ε in the fiber direction (Figure 4). The model findings proved the effectiveness of the proposed design in concentrating ε along the longitudinal axis of the optical fiber with the maximum value attained where the FBG sensor is located during both breathing and cardiac activity.

### 2.3. The Smart Patch Fabrication Process

**Figure 5:**
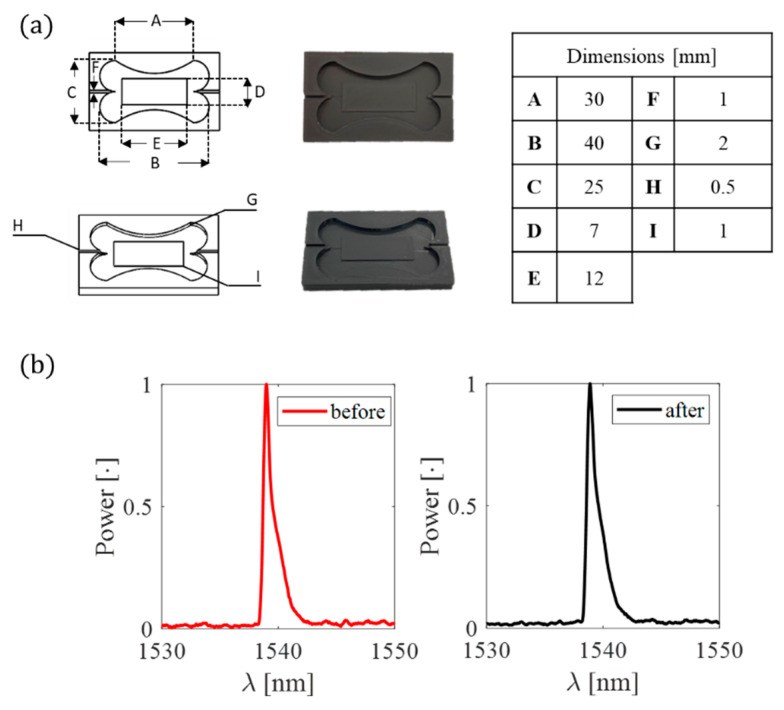
(**a**) Mold geometrical dimensions and (**b**) the spectrum before (red line) and after (black line) the silicone pouring.

After the patch shape validation, a mold was firstly designed using Solidworks^®^ software and then 3D printed in polylactic acid (PLA) by Ultimaker 2^+^ (Ultimaker, Utrecht, The Netherlands) for conferring the chosen shape to the flexible matrix (Figure 5).

**Figure 6:**
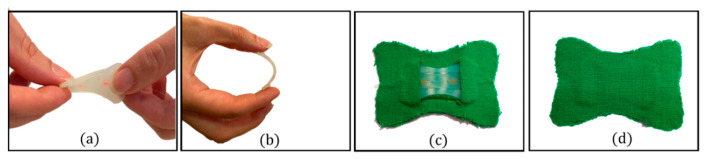
(**a**) Twisting and (**b**) bending of the silicone layer embedding the FBG sensor; (**c**) the smart patch backend and (**d**) frontend.

The FBG sensor was placed inside the mold before the polymer preparation. A pretension was applied to the fiber ends, part A of the silicone was equally mixed with part B, and the mixture was degassed and poured into the mold to cover the grating inside. After a curing time of 4 h, the silicone rubber vulcanized, and the flexible sensor was taken out of the mold. During the fabrication process, the power spectrum of the pretensioned FBG inside the mold before the silicone pouring and after the silicone curing was collected (see Figure 5b red and black lines, respectively). No spectrum changes occurred in central wavelength (λ_B_) and shape. Finally, the soft sensor was sandwiched between the two fabric liners apart from the sensing part (see Figure 6) The overall dimensions of the smart patch are 40 mm × 25 mm × 2 mm.

The silicon rubber (i.e., Dragon Skin^TM^ 20) served as an encapsulating layer for the FBG sensor to improve the system adherence to the skin and robustness (see Figure 6a,b). The fabric liners allowed to enlarge the flexible matrix contact surface with the skin and facilitated the skin–sensor coupling (see Figure 6c,d).

## 3. The Smart Patch Metrological Assessment

The smart patch was developed to be mounted on the skin to monitor HR and RR, starting from detecting the chest wall deformations due to breathing and heart beating. Regular breath-to-breath and beat-to-beat patterns induce repetitive tensions and compressions on the multi-layered structure, inducing an ε field on the FBG into the silicone layer and, consequently, a Δλ_B_. The stronger the polymer-fiber bonding strength, the better the ε transmission [49]. To better investigate the metrological properties of the developed biosensor, we firstly analyzed the response to ε to obtain $K_{\varepsilon }$ Then, we also evaluated the influence of T and relative humidity (RH) on the sensor output. This analysis was carried out since the smart patch was developed to be attached to the chest epidermis; hence, body ΔT and/or sweating may occur and potentially affect the sensor functioning. Finally, considering the periodic pattern of cardiorespiratory activity, we also evaluated the hysteresis error (h_err_) at velocities mimicking typical RR and HR values.

### 3.1. Strain Response

**Figure 7:**
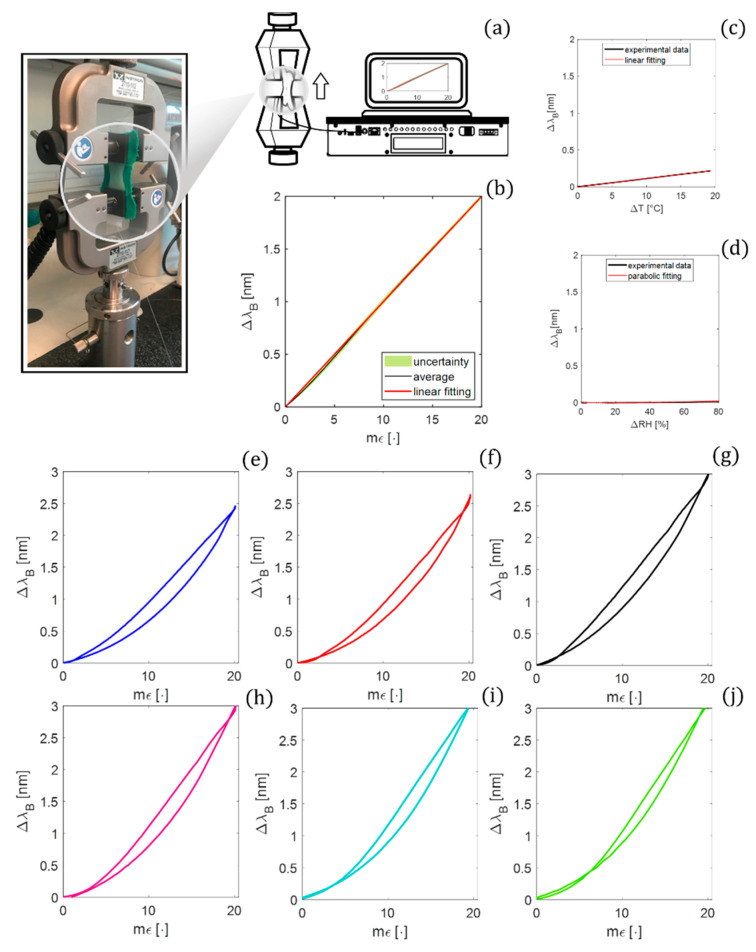
(**a**) Experimental setup for $K_{\varepsilon }$ investigation with a zoom on the smart patch between the grips; (**b**) the calibration curves Δλ_B_ vs. ε; (**c**) Δλ_B_ vs. ΔT; (**d**) Δλ_B_ vs. ΔRH; the hysteresis cycle with the $h_{\mathrm{err}}^{\max}$ at velocities mimicking RR of (**e**) 12 apm; (**f**) 24 apm; (**g**) 36 apm and HR of (**h**) 60 bpm; (**i**) 90 bpm, and (**j**) 120 bpm.

A tensile testing machine (Instron, mod. 3365) was used to carry out a static assessment of the proposed biosensor by positioning the patch between the lower and upper grips (see Figure 7a).

A low displacement rate (i.e., 2 mm·min^−1^) was applied in order to guarantee a quasi-static condition starting from an initial length (l_0_) of 25 mm. To cover the ε range that the sensor could experience in the scenario of interest, a maximum strain ($\varepsilon_{\max}\%$) of about 2 % (i.e., 0.5 mm) was applied.

The output of the tensile machine was recorded at a sampling frequency of 10 Hz, while the Δλ_B_ values of the FBG by an optical spectrum interrogator (si255 based on HYPERION platform; LUNA Inc.) were at the sampling frequency of 100 Hz. This mechanical test was performed ten times to investigate the repeatability of the system response to the applied ε. The raw data were processed through a custom algorithm to extract the calibration curve (Δλ_B_ vs. ε). The average Δλ_B_ values over the ten tests were computed. Then, the expanded uncertainty was estimated as the standard uncertainty multiplied by the coverage factor (k = 2.776), considering a t-Student distribution with nine degrees of freedom and a confidence level of 95%. The calibration curve was obtained by fitting the experimental data with the best polynomial curve (see Figure 7b). The results fall on a linear trend (R^2^ > 0.99); hence, the $K_{\varepsilon }$ value was considered equal to the angular coefficient of the fitting line (i.e., 0.10 nm mε^−1^).

### 3.2. Influence of Temperature and Relative Humidity

The influence of the physical quantities of T and RH was investigated to strengthen the usability of the developed patch as a strain sensor.

For the analysis of T influences, the biosensor was placed within a laboratory oven (PN120 Carbolite Gero^®^, Derbyshire, UK) and exposed to T changes (ΔT) of ~20 °C (i.e., from 26 to ~46 °C). The test was carried out as follows: once the maximum value of T was reached, the oven was switched off, and data were collected until T reached the ambient temperature (for approximately 7 h) to guarantee a static assessment. Reference values of T were recorded by a thermistor (EL-USB-TP-LCD, EasyLog, Lascar Technology, Whiteparish, UK) and the output of the smart patch by an FBG interrogator (FS22, HBM FiberSensing, S.A., Moreira, Portugal). A sampling frequency of 1 Hz was set for both the devices.

The best-fitting curve was computed to model the Δλ_B_ vs. ΔT. A linear response was found with a $K_{T}$ of 0.01 nm·°C^−1^, which is one order of magnitude lower than $K_{\varepsilon }$ (see Figure 7c).

To investigate the effect of RH on the smart patch response, the proposed system was placed inside a custom climatic chamber and exposed to quasi-static RH changes. Once the level of RH reached 100%, dry air was forced inside the chamber at a flow rate of 1 L·min^−1^ to slowly lower RH to ~20%. A capacitive-based RH sensor (HIH 4000-002, Honeywell International Inc., Morristown, NJ, USA) was connected to a data acquisition board (NI DAQ USB-6009, NI Instruments, Austin, TX, USA), and a LabVIEW interface ad-hoc developed for the real-time tracking the changes in the level of RH inside the chamber (ΔRH of 80%), Both Δλ^B^ and RH values were collected at 100 Hz.

The best-fitting curve was computed to model the Δλ_B_ vs. ΔRH. A parabolic response described by $y=0.004x^{2}+0.0070x+0.0035$ was found (see Figure 7d) with a sensitivity to RH ($K_{\mathrm{RH}}$) ~0.0002 nm·RH^−1^ evaluated as:

(3) $$K_{\mathrm{RH}}=\frac{{\Delta \lambda }_{B}\left({\mathrm{RH}}^{\max}\right)-{\Delta \lambda }_{B}\left({\mathrm{RH}}^{0}\right)}{{\mathrm{RH}}^{\max}-{\mathrm{RH}}^{0}}$$

### 3.3. Hysteresis Error

The tensile testing machine (Instron mod. 3625) was used to perform the following analysis. Considering the physiological dynamics of the respiratory and cardiac activity, ten hysteresis cycles were performed at three different speeds mimicking RR of 12 respiratory acts per minute (apm), 24 apm, and 36 apm, and three different speeds mimicking HR of 60 beats per minute (bpm), 90 bpm, and 120 bpm, respectively. The outputs of the biosensor and the tensile testing machine were collected at the sampling frequency of 100 Hz. The hysteresis error (h_err_) of each cycle was calculated by:

(4) $$h_{\mathrm{err}}=\frac{{\left({{\Delta \lambda }_{B}}^{a}-{{\Delta \lambda }_{B}}^{d}\right)}^{\max}}{{{\Delta \lambda }_{B}}^{\max}}\cdot 100$$

with (${{\Delta \lambda }_{B}}^{a}-{{\Delta \lambda }_{B}}^{d}$)^max^ being the maximum value of the difference between wavelength changes recorded during the ascending and the descending phases at the same input (i.e., ε expressed in terms of mε) and ${{\Delta \lambda }_{B}}^{\max}$ being the maximum ${\Delta \lambda }_{B}$ value.

The maximum h_err_ ($h_{\mathrm{err}}^{\max}$) among the 10 cycles for each mimicked RR and HR value is listed in Table 2 and plotted in Figure 7e–g or Figure 7h–j, respectively.

## 4. The Smart Patch Feasibility Assessment of Healthy Volunteers

### 4.1. Experimental Protocol and Setup

A field study on healthy volunteers with no history of cardiorespiratory diseases (preclinical trial titled Smart Textile—Università Campus Bio-Medico di Roma, protocol number ST-UCBM 27.2(18).20 OSS granted by the Ethical Committee of Università Campus Bio-Medico di Roma Rome, Italy) was conducted to assess the feasibility of the smart patch to monitor HR and RR. The enrolled subjects (Ss) were nine adults, S1·S9 (60% males and 30% females), with an age of 28 ± 5 years old and a BMI of 24.2 ± 2.6. All the human characteristics are expressed as mean ± standard deviation.

**Figure 8:**
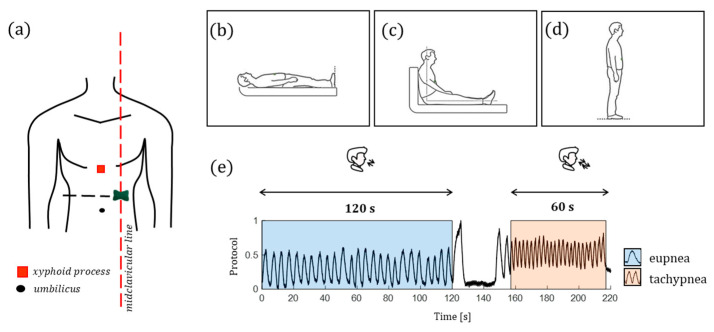
(**a**) The sensor positioning and the assumed body positions: (**b**) supine; (**c**) sitting, and (**d**) standing; (**e**) the experimental protocol: the 120 s of eupnea in the blue-colored background and the 60 s of tachypnea in the orange-colored background.

The biosensor was directly patched on the skin on the left sternum along the midclavicular line considering the midpoint between the xiphoid process and the umbilicus (Figure 8). Each volunteer was invited to perform three identical trials. The sole difference consisted in the body position assumed by the participants during each trial: the first trial was carried out in a supine position (Figure 8b), the second one in a sitting position (Figure 8c), and the third one in a standing position (Figure 8d). The performed protocol consists of a two-stage procedure for each trial: 120 s of eupnea separated by 60 s of tachypnea. All the volunteers performed an end-expiratory apnea of ~15 s after a deep breath to better discriminate these stages. Then, the volunteer performed two breaths to recover from the breath-hold and started the tachypnea stage (Figure 8e). At the end of tachypnea, each volunteer was invited to change the body position (the first time from laying down to sitting up and the second time from sitting up to standing up). The subsequent trial began once the participant was ready to start.

**Figure 9:**
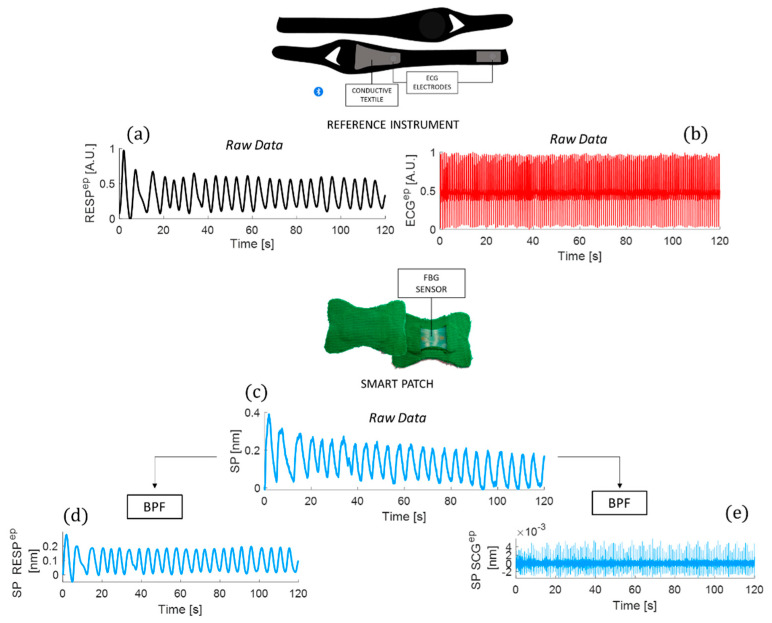
The raw signals recorded during a trial: (**a**) the respiratory trace—RESP^ep^; (**b**) the ECG^ep^; (**c**) the smart patch raw signal (SP) recorded during a trial; (**d**) the band pass filtered (BPF) respiratory signal—SP RESP^ep^; and (**e**) the BPF cardiac signal—SP SCG^ep^. The ep apex refers to eupnea stage. By way of example, the 120 s of eupnea on a male volunteer while laying down is shown.

During the testing procedure, the reference breathing and ECG waveforms (Figure 9a,b) were acquired using a commercially available chest strap (Zephyr^TM^ performance systems, Medtronic, The Netherlands) as a benchmark. Simultaneously, the smart patch output in Figure 9c was recorded using an optical interrogator (si255, Hyperon platform, LUNA Inc., Roanoke, VA, USA).

### 4.2. Data Analysis and Results

All the collected data were analyzed in the MATLAB^®^ environment. Raw data were synchronized, and the smart patch signal, the reference respiratory signal, and the ECG signal were split into the eupnea-related traces and tachypnea-related traces. Then, three main steps were performed to extract RR and HR values:

- The signal filtering and windowing;
- The power spectral density (PSD) analysis;
- The statistical analysis.

#### 4.2.1. The Signal Filtering and Windowing

A first-order Butterworth bandpass filter (BPF) with a lower cut-off frequency of 0.05 Hz and a higher cut-off frequency of 0.5 Hz was implemented to better emphasize the respiratory activity contributions in the smart patch signal (see Figure 9d). The same BPF was applied to the respiratory traces recorded by the reference instrument. For the cardiac investigation, the raw signal recorded by the smart patch was filtered by a first-order Butterworth BPF with a lower cut-off frequency of 10 Hz and a higher cut-off frequency of 30 Hz to emphasize the signal related to the cardiac contributions (referred to as SCG signal in Figure 9e) masked by the respiratory one. The ECG trace was BPF with a lower cut-off frequency of 0.7 Hz and a higher cut-off frequency of 2 Hz. Finally, the SCG signal was enveloped to better emphasize the peaks related to the aortic valve opening (AO).

#### 4.2.2. The Power Spectral Density Analysis

**Figure 10:**
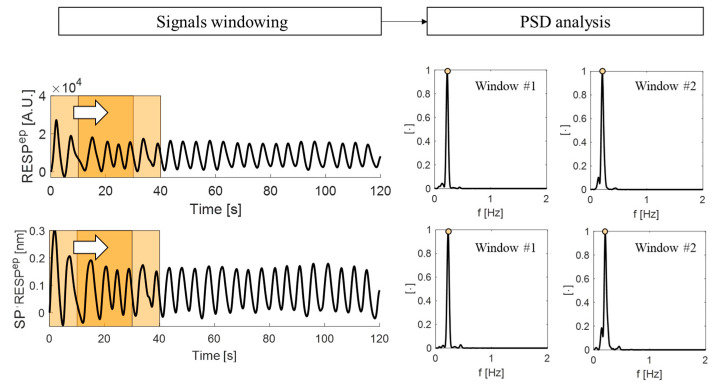
The signals windowing performed over the respiratory signals (reference trace RESP^ep^ and SP RESP^ep^) recorded during eupnea in supine position. Two consecutive 30 s windows with an overlap of 10 s are highlighted (orange squares). For each window, the PSD spectrum is computed.

The estimation of RR and HR values from the filtered signals was performed in the frequency domain following Welch’s method to estimate the power spectral density (PSD) and by implementing an overlapping sliding window analysis [50]. For the respiratory signals, the overlap between adjacent windows was set to 10 s, while it was set to 5 s for the cardiac signals. This setting considers differences between typical respiratory and cardiac timing. For each overlapping window, the PSD was estimated, and the dominant frequency of each spectrum was evaluated as the frequency value at which the maximum peak of the spectrum is located. Figure 10 shows an example of signal windowing with the corresponding PSD spectrum.

**Figure 11:**
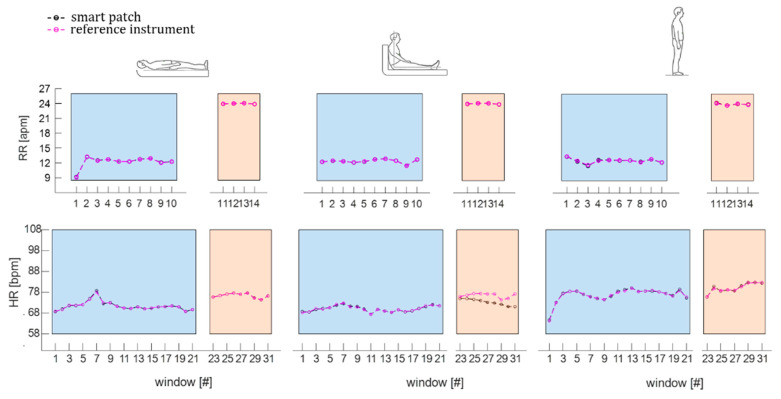
The window-by-window RR values, estimated during eupnea (light blue backgrounds) and tachypnea (light orange backgrounds) in supine, sitting, and standing positions, respectively, and the window-by-window HR values, estimated during eupnea (light blue backgrounds) and tachypnea (light orange backgrounds) in supine, sitting, and standing positions.

Therefore, the RR and HR values estimated by the smart patch and the reference system were obtained from the PSD dominant frequency and expressed in apm and bpm, respectively. The windowed values of RR and HR of a subject (i.e., S1) in the three assumed positions during eupnea and tachypnea are shown in Figure 11.

#### 4.2.3. The Statistical Analysis

Finally, the agreement between the values measured by the smart patch and the reference instrument was evaluated in terms of percentage error (err) and by performing a Bland–Altman analysis in terms of the mean of difference (MOD) and level of agreements (LOAs) [51].

To estimate the err values for RR monitoring, the difference in terms of breaths between RR values estimated by the smart patch (RR^SP^) and the reference instrument (RR^ref^) was evaluated per each subject in each position and averaged over the participants. Results showed comparable performance of the smart patch in RR monitoring during supine (err value of 0.05 apm in eupnea and 0.07 apm in tachypnea) and sitting (0.05 apm in eupnea and 0.08 apm in tachypnea) positions. In comparison, err values increased in standing position (0.10 apm in eupnea and 0.14 apm in tachypnea).

Regarding the estimation of err values for HR monitoring, the results showed the lowest discrepancy in terms of HR values in the supine position (err value of 0.32 bpm in eupnea and 0.23 bpm in tachypnea) followed by standing (0.63 bpm in eupnea and 0.74 bpm in tachypnea) and sitting positions (0.46 bpm in eupnea and 4.49 bpm in tachypnea).

**Figure 12:**
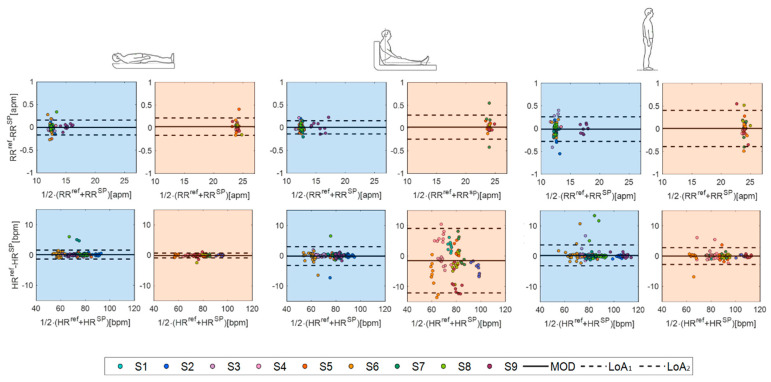
Bland–Altman plots: MOD (black line) and LOAs (black dotted lines) in eupnea (light blue backgrounds) and tachypnea (light orange backgrounds) in supine, sitting, and standing positions.

Finally, we performed a Bland–Altman analysis to deeply investigate the performance of the proposed system in cardiorespiratory monitoring (Figure 12). The smart patch showed high performance in RR estimation in all the three body positions assumed during both eupnea and tachypnea (Figure 12). The best accuracy in RR estimation was obtained in supine position: a MOD of 0.003 apm with a total span (LOA_1_-LOA_2_) of 0.330 apm in eupnea and a MOD of 0.027 apm with LOA_1_-LOA_2_ of 0.355 apm. Focusing on HR measurements, again, the smart patch reached again the best performance in supine position, followed by standing and sitting positions, showing a MOD of 0.12 bpm with an LOA_1_-LOA_2_ of 2.93 bpm in eupnea and a MOD of 0.79 bpm with LOA_1_-LOA_2_ of 1.59 bpm in tachypnea.

**Figure 13:**
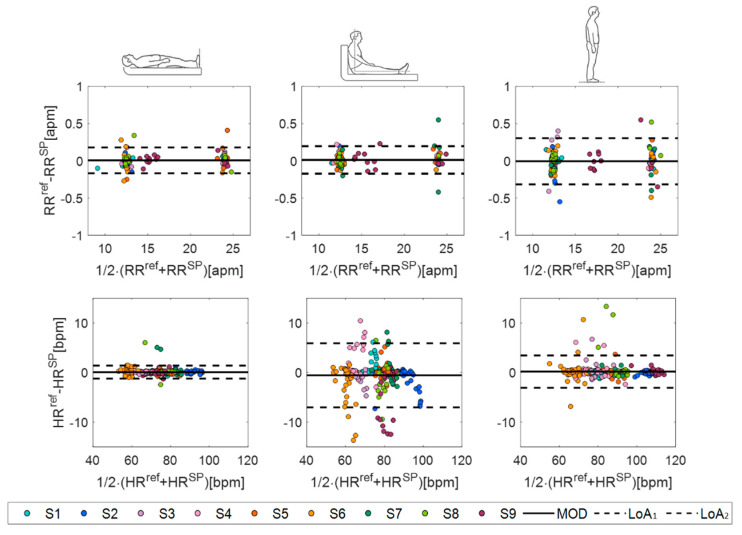
Bland–Altman plots: MOD (black line) and LOAs (black dotted lines) related to RR and HR in supine, sitting, and standing positions.

To better emphasize the influence of body postures on the smart patch performances, data collected during eupnea and tachypnea were grouped per position (see Bland–Altman plots in Figure 13). As expected, the highest performance in both RR and HR monitoring was found in the supine position, as summarized in Table 3.

## 5. Discussion

The soft skin-interfaced smart patch used in this study is a less invasive and more comfortable solution for cardiorespiratory monitoring compared to the gold standard techniques and state-of-the-art solutions. We demonstrated the high capability of the proposed biosensor of monitoring both RR and HR using a single sensing modality (i.e., an FBG sensor), very compliant with the skin, and usable long-term. As confirmed by a model simulation, the unique shape, form factor, and flexible mechanics of the proposed patch facilitated the strain concentration along the encapsulated grating (as shown by the computational model), improving the signal quality. We proposed an innovative smart patch to foster a major advance over existing flexible wearable sensors for cardiorespiratory monitoring. In the literature, wearables have been often used to detect RR exclusively, and patches have been little explored [52]. In a few cases, HR has been monitored by using single sensing modalities, but the highest measurement accuracy was reached during breath-hold stages when the user, often laid down, is invited to stop to breathe at one point (usually for 10 s up to ~30 s) [25,26,31,32].

The distinctive features of the proposed smart patch can overcome this issue by promoting HR monitoring even during breathing, over longer periods of time, and under different body positions. Indeed, the high potentiality of the smart patch confirmed by trustworthy computational models was further assessed on healthy volunteers while assuming those positions mimicking common resting conditions (e.g., watching tv, reading a book, sleeping).

Although the literature has reported a few studies based on the assessment of wearables’ capabilities for cardiorespiratory monitoring [31,53], no one has proposed skin-interfaced patches based on FBG technology and compared the system performances under various typical daylong postures using a single sensing modality. In general, the available studies discuss the feasibility assessment when a specific body position is assumed, typically when the user is supine, including multiple types of sensors for achieving better performance. Furthermore, wearable systems are often tailored to male anthropometry; hence, their usage on females is not recommended since it can cause a deterioration in performance. Otherwise, as confirmed by our results, the smart patch design allows for an easy sticking of the sensor on a small area of the chest surface regardless of sex-specific associations of anthropometric measurements.

The primary limitation of this work is related to the small sample size. Another limitation is that data collected during the sitting position suggest that the sensor performance can be hampered by a weak adhesion of the skin–sensor interface. When lying down (the first position assumed by each volunteer in this study), gravity pulls the abdominal contents downwards, the stomach goes flat, and the sensor firmly adheres to the skin. Then, from lying down to sitting up (the second position assumed by each volunteer), the abdominal area is not completely flat. Therefore, some changes may occur at the sensor–skin interface and affect the sensor response. This condition can explain the performance reduction in monitoring HR while sitting, especially during tachypnea. Otherwise, when laying down or sitting up, the body is stretched out, and the abdominal fat is extended over a larger area, leading to conformal contact of the patch to the chest surface. The smart patch assessment in static conditions can also be considered a study limitation. However, the high clinical relevance of resting RR and HR values motivated our experimental protocol [10]. Future works will address this lack by exploring the system accuracy while performing daily life activities and after physical exercises. In this scenario, the encumbrance of the interrogation unit may reduce the usability of the smart patch. However, the recent on-market availability of small FBG interrogators may help in overcoming this shortage and facilitate the use of the smart patch during dynamic tests. These working conditions will allow us to better investigate the smart patch’s capability of detecting rapid changes in HR and RR caused by the performed activity and its power to remain conformally interfaced with the skin despite sweat and tangential stresses caused by both body movements and cloth frictions.

## 6. Conclusions

In conclusion, the smart patch presented in this study introduces a highly miniaturized and stretchable biosensor, which can be readily applied for cardiorespiratory monitoring in both clinical (such as on bedridden or wheeled patients and during MR examination) and real-life scenarios (e.g., while watching TV, reading a book, working at the desk, and sleeping). In such a context, standard monitoring technologies are not reachable or convenient. We envision that using the presented approach based on fiber optics and soft packaging together with the FBG interrogation advancements in terms of performance and cost, a new class of highly flexible wearables for non-invasive cardiorespiratory monitoring could be harnessed into a broad range of home monitoring solutions in the future.

## Figures and Tables

**Table 1:** Values of model parameter employed in numerical simulation.

Model Parameters	Value
Skin geometry model	200 × 120 × 5 [mm]
Stretch boundary condition	(2, 4, 6, 8, 10, 12, 14) [mm]
Shape function r(x,t)	−0.5·x^2^
Skin E and ν values	(130 × 103, 0.49) [Pa, -]
Fabric liner E and ν	(2 × 104, 0.3) [Pa, -]
Dragon Skin silicone E and ν	(45 × 105, 0.49) [Pa, -]

**Table 2:** Values of $h_{\mathrm{err}}^{\max}$ expressed in percentage.

RR and HR Values		$h_{\mathrm{err}}^{\max}$ [%]
RR Value	12 apm	14.08%
	24 apm	15.84%
	36 apm	19.32%
HR Value	60 bpm	22.38%
	90 bpm	23.94%
	120 bpm	24.78%

**Table 3:** Bland–Altman summary results.

	Supine		Sitting		Standing	
	RR [apm]	HR [bpm]	RR [apm]	HR [bpm]	RR [apm]	HR [bpm]
**MOD**	0.0059	0.071	0.012	−0.5515	−0.006	0.1757
**LOA_1_**	0.1796	2.6174	0.1983	5.9152	0.3046	3.4398
**LOA_2_**	−0.1679	−1.3797	−0.1739	−7.0182	−0.3167	−3.0885

## Data Availability

The data sets generated during and/or analyzed during the present study are available from the corresponding author upon reasonable request.
